# Leaf Nutrient Status of Commercially Grown Strawberries in Latvia, 2014–2022: A Possible Yield-Limiting Factor

**DOI:** 10.3390/plants12040945

**Published:** 2023-02-19

**Authors:** Anita Osvalde, Andis Karlsons, Gunta Cekstere, Laura Āboliņa

**Affiliations:** Institute of Biology, University of Latvia, LV-1004 Rīga, Latvia

**Keywords:** *Fragaria x ananassa*, leaf analysis, sulphur, calcium, zinc, copper

## Abstract

The present study was carried out to evaluate the leaf nutrient status of commercially grown strawberries in Latvia during 2014–2022. The results of N, P, K, Ca, Mg, S, Fe, Mn, Zn, Cu, Mo and B in 200 strawberry leaf samples from different strawberry-producing farms were analysed over three periods: 2014–2016, 2017–2019, and 2020–2022. According to leaf analyses, plant fertilization was only partly well managed by the growers. During the research period, strawberries in Latvia were generally sufficiently supplied with N, P, K, Mg, Fe, Mn, Mo, and B, while the level of Ca, S, Zn, and Cu was considered low. The deficiency of these nutrients was characteristic for more than 50% of the samples. Since Ca, S, Zn, and Cu are essential for berry formation and quality and contribute to stress resistance; their deficiency could be one of the limiting factors for strawberry yield. The significant positive correlations found between nutrients, including deficient ones, confirmed their close relationship in the uptake process and the importance of sufficient supply. The results clearly indicated that fertilization could currently be an issue that limits the strawberry harvest in Latvia, and adequate provisions of Ca, S, Zn, and B should be the main focus.

## 1. Introduction

Global strawberry (*Fragaria x ananassa* Duch.) production has increased by nearly 40% over the past 10 years to meet growing demand. Today, the largest world producer is China, followed by the USA and Mexico [1]. In the European Union, Spain and Poland are among the top 10 countries in strawberry production. The strawberry is also one of the most popular and commercially important berry crops in Latvia. However, from 2013 to 2021, the harvested area has not increased and amounts to approximately 500 ha. Although the average strawberry yield has fluctuated slightly over the recent 5 years, it is still trending downward [2]. As a result, annual production also decreased from 2015 to 2021 (from 1343 t to 1100 t). In 2019, Latvia ranked 76th among other countries in strawberry yield with 2.43 t ha^−1^. It is considerably lower than other countries of the Baltic Sea region as Germany (10.9 t ha^−1^), Sweden (8.3 t ha^−1^), Norway (6.9 t ha^−1^), Denmark (5.7 t ha^−1^), Finland (4.1 t ha^−1^), and Poland (3.7 t ha^−1^), but similar to the other Baltic States, such as Estonia (2.5 t ha^−1^) and Lithuania (2.4 t ha^−1^) [1]. The amount of strawberries grown in the country comprises only a small fraction of the World Health Organization’s recommended 400 g of fruits and vegetables per day.

In general, there are different reasons for low berry yields. As the majority of plantations in Latvia are located in the open field, strawberries are exposed to adverse weather conditions, such as spring frosts, drought, and heavy rains during the summer [3]. In recent years, high-yielding strawberry varieties and new cultivation technologies have been introduced in Latvia [4,5]. Growing in low and high tunnels, as well as in greenhouses, extends the berry season and largely prevents the impact of unfavourable weather conditions [6,7]. However, currently, only less than 5% of harvested strawberries are produced in greenhouses [2].

The growth, yield, and quality of strawberries do not only depend on meteorological conditions but also on various agricultural treatments used during the growing season. Since the plants have a relatively shallow root system, and the formation and ripening of fruits occur shortly, about 20–40 days after pollination [8], sufficient and precise fertilization is very important. Complete and balanced nutrition has always been the basis of plant productivity due to the direct involvement of mineral nutrients in the formation of plant biomass. According to different studies, strawberries are susceptible to nutrient-related disorders [9,10,11]. Therefore, regardless of the type of cultivation and varieties used, appropriate plant nutrition is very important for high berry yield and quality [12,13,14]. In addition, fertilization is also closely related to economic and environmental aspects.

In monitoring plant nutrient status, tissue testing is considered to be one of the best tools for the management of the fertilization program. The obtained data are also comparable with established target concentrations for healthy strawberry leaves that have been reported by different authors worldwide [15,16,17,18]. Although experimental research on various issues relating to strawberry cultivation is ongoing worldwide, there is very little published data on the actual mineral nutritional status of commercially grown strawberry plantings [19,20]. There is generally no such data for the Baltic region. So far, only a few fragmentary studies have been conducted in Latvia on strawberry fertilization [21,22,23,24]. More information and knowledge are needed on existing fertilization practices and their impact on/relationship with berry yield and quality to solve the causes of low strawberry yields.

Therefore, this study was carried out to evaluate the leaf nutrient status of commercially grown strawberries in Latvia during 2014–2022. We aimed to clarify whether the level of nutrient supply and possible imbalances could be limiting factors for the strawberry harvest in Latvia.

## 2. Results and Discussion

Leaf analysis is considered a highly effective approach for monitoring the nutritional status of crop cultures and addressing potential deficiencies. They not only contribute to the optimization of the yield and its quality but also protect against the excessive entry of nutrients into the environment and unnecessary expenses [8,18]. To characterize the nutrient status of strawberries in Latvia, the concentration of 12 essential nutrients (N, P, K, Ca, Mg, S, Fe, Mn, Zn, Cu, Mo, and B) were estimated in strawberry leaf samples from commercial fields. Overall, the chemical results revealed the low heterogeneity of leaf macronutrient concentrations throughout the study period from 2014 to 2022 (Table 1). Higher variability was found for micronutrients. Among them, the highest coefficient of variation was characteristic of Mn and Mo. This could largely illustrate the differences that exist in soil pH levels for strawberry soils in Latvia. The availability of Mn to plants is strongly promoted by a low soil pH, while Mo by high soil pH, even if the content of these elements does not differ from soil to soil [25]. The application of foliar fertilizers shortly before leaf sampling may also be considered in some cases.

In general, strawberry cultivars can be categorized into three types based on their response to photoperiod: June-bearing, day-neutrals, and ever-bearers. June-bearing cultivars are the most common and most widely grown worldwide [15]. Various early-season, mid-season, and late-season June-bearing varieties are cultivated to extend the harvest time. Most nutrient adequacy ranges refer to June cultivars and to the harvest period when leaf nutrient concentrations are more stable [15,17,18]. As leaf nutrient concentrations change throughout the season, certain sources in the literature also indicate pre-harvest sufficiency levels, which usually have slightly higher N, K, and Mg concentrations [26]. The recommendations for day-neutral strawberries are mainly developed for the cultivar ‘Albion’, which is widely grown in Australia and the USA [16,18,20]. These recommendations generally show very similar ranges to those recommended for June-bearing strawberries, except for lower Zn and Cu but higher Mn concentrations in the leaves. In the case of our study, all cultivars were June-bearing, and the leaf sampling time from June to August generally corresponded to the harvest period. Therefore, the specified nutrient-sufficiency ranges of strawberry leaves (Table 1) corresponded to these conditions.

**Figure 1:**
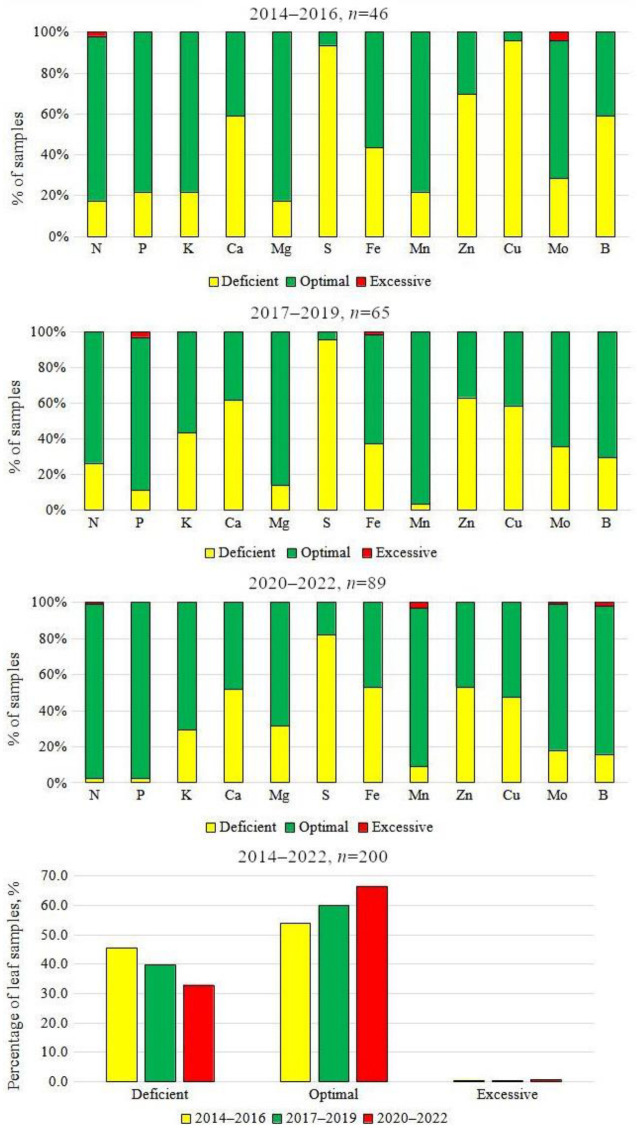
Distribution of strawberry leaf samples from commercial plantings in different nutrient supply levels in Latvia, 2014–2022.

A survey on the nutrient status of commercially grown strawberries revealed that the mean leaf N, P, K, Ca, Mg, Fe, Mn, Mo, and B levels were within the literature-reported [16,17,18,26] sufficiency range for strawberries throughout the whole study period (Table 1). Although a small positive trend was found in the supply of strawberries with nutrients: the percentage of total optimal concentrations in the leaves increased from 54% in 2014–2016 to 67% in 2020–2022: more than a third of the indices did not reach the desired levels (Figure 1).

Of the macronutrients, only the mean concentration of S was found to be below the recommended 0.20%. Although the study showed a low level of S in the strawberry leaves for 80–95% of samples during the whole study period (Figure 1), recently (2020–2022), there has been a slight tendency for the percentage of optimal results to increase. Sulphur is essential for many plant functions as a structural component of proteins, peptides, and various enzymes [25]. A sufficient S supply has been found to improve fruit quality and aroma and to promote the plant’s resistance to environmental and pathogen stress [12,27,28]. In addition, decreased S can create an imbalance with N in plant metabolism. Nitrogen-to-S ratios greater than 18:1 can result in the poor utilization of N and S even when tissue levels of these nutrients are adequate [29,30]. In Latvia, more than 65% of the strawberry samples had N:S ratios greater than 18:1 for the period from 2014 to 2019, when the mean S concentration was below the critical value of 0.15%, as reported by Bottoms et al. [13]. In the period from 2020 to 2022, the number of such samples decreased to 50%, along with the improvement in S supply.

In general, there was a significant positive correlation between S and N, P, and K (0.419 > *r* < 0.566, *p* < 0.05) in strawberry leaves (Table 2). No significant negative correlations with any of the nutrients were found. Various researchers have reported synergistic interactions between the N and S of different crop plants, which is mainly explained by the central role of S and N in protein synthesis [31,32,33]. In addition to N, a synergistic effect of S with P and K was also previously confirmed [33]. Thus, sufficient S supply is essential for the optimal accumulation of other nutrients required for vital plant growth and development.

A positive correlation between S and N, P, and K also indirectly indicates the use of sulphur-containing complex fertilizers in strawberry fertilization. However, it should be noted that S available to plants in the soil solution in the form of sulphate (SO_4_^2−^) is highly mobile, with only weakly held on colloidal particles, and therefore is easily leached, especially from sandy, coarse-textured soils with low organic matter content [34]. In addition, a huge reduction in industrial emissions has resulted in a massive decrease in atmospheric S over the last decades [27,35]. Therefore, attention should be paid to the optimization of the S supply for strawberries in Latvia. This is particularly important for field-grown strawberries, as optimizing S nutrition is necessary for plants to ensure strong cell walls and promote resistance to dehydration and diseases. The deficit of plant-available S could be effectively mitigated by developing site-specific S supply strategies under different soil conditions. Soil fertility management using organic fertilizers, sulphate-containing chemical fertilizers, and/or slow-release S fertilizers should be recommended as an effective way to ensure that S is not the limiting nutrient for high yields.

Similar to S, calcium is also considered a nutrient that undeniably affects both plant growth and development, responses to biotic and abiotic stresses, as well as berry quality. Although the average Ca content of strawberry leaves was at the lower end of the recommended range of 0.70–1.5% [15,17], there were a large number of analyses with low values. Our study showed that approximately 50–60% of the strawberry leaf samples in Latvia were not provided with a sufficient Ca level. In addition, several recommendations specify a higher level of 1.0% Ca—as the minimum required Ca content for strawberry leaves [16,18]. As Ca is a key nutrient involved in cell division and the maintenance of cell permeability and cell integrity [25], a sufficient Ca concentration in plant tissues is always a quality factor and may have various beneficial effects, including fruit firmness, lower fruit acidity, and higher fruit quality after harvest [12,36,37]. Unlike S, calcium deficiency in plants is rarely a direct result of a lack of Ca in the soil and, therefore, may not be easily corrected by simply increasing Ca fertilization. Ca deficiency more often is caused by the limited allocation of Ca when driven by transpiration. As Ca moves only in the xylem, cool, wet, or cloudy weather, as well as insufficient soil moisture, can inhibit Ca uptake. In such cases, foliar Ca supplementation is advisable. The results of various studies have shown that the foliar application of Ca reduced the frequency of disorders (albinism, Botrytis fruit rot) and improved the qualitative and quantitative characteristics of fruits, resulting in a higher marketable yield of strawberries [12,36,38,39]. However, opposite or contradictory results have also been reported for the foliar application of Ca fertilizers. Thus, the foliar application of natural calcite had no statistically significant effect on the strawberry quality and did not reduce pest and disease damage [23]; sprays with calcium nitrate did not significantly increase the fruit yield [38]. According to Vance et al. [40], targeted Ca applications (Ca chloride, Ca silicate, Ca chelate, and Ca acetate) at the rates currently recommended were not effective at increasing fruit or leaf Ca concentrations or altering fruit quality at harvest and during storage. It is possible that Ca treatment can only be effective when tissue levels are below the sufficiency level. Therefore, when fertilizing strawberries in Latvia, attention should be paid not only to adequate S and Ca provision but also to the methods of their application.

Micronutrients play a vital role in the production of fruit crops, and their deficiency can greatly affect fruit quality. In Latvia, of the 200 strawberry leaf samples analysed from 2014 to 2022, Zn deficiency was found in 50–70% of cases. Among micronutrients, horticultural crops suffer from Zn deficiency worldwide, especially in calcareous soils, but also in light sandy and peaty soils [41]. Such agricultural soils are typical for Latvia as well [42]. Studies on different strawberry cultivars and most recommendations for sufficiency levels indicated that Zn deficiencies in strawberries were associated with leaf concentrations below 20 mg kg^−1^ [9,17,18]. The fact that the mean values of Zn in strawberry leaves were on the lower limit and more than half of the samples were deficient indicates the need to optimize the Zn supply of commercially grown strawberries in Latvia. Since Zn plays an essential role in pollination, fruit set, and the improvement of fruit quality parameters, in the case of its deficiency, a decrease in fruit formation can be predicted. In addition, Zn is important in disease resistance and healthy plant growth [28]. Various studies suggested that the foliar application of Zn should be included in strawberry cultivation for enhanced plant growth and yield [43,44,45].

Correlation analysis revealed that concentrations of Zn, N, P, K, and S in strawberry leaves were significantly positively correlated (0.536 > *r* < 0.680, *p* < 0.05) throughout the study period. N, P, K, and S are essential in plant life processes such as respiration and photosynthesis, osmotic regulation, energy transfer, protein synthesis, enzyme activation, and many others [12]. According to Prasad et al. [46] and Tohidloo et al. [47], the positive significant correlation among N, P, and K is necessary for better strawberry development which determines the balance between vegetative growth, fruit quality, and reproductive processes. Therefore, positive interactions between Zn and these nutrients indicate the contributing role of a higher macronutrient supply in the development of the plant, including the root system, which also promotes the absorption of micronutrients. The close relationship between nutrients in their uptake process was also confirmed by Salman et al. [48], who reported that the foliar application of Ca, Zn, and B exhibited significantly increased macronutrient (N, P, K, Ca) and micronutrient (Zn, B, Fe) contents in strawberry leaves, and consequently, fruit yield.

Copper is one of the essential nutrients that are necessary for optimal plant growth and reduced susceptibility to fungal and bacterial diseases [12,49]. In comparison with other micronutrients, there were almost no data on Cu deficiency for strawberries. Although Cu acts as an important cofactor for several enzymes and also plays an important role in respiration and photosynthetic metabolism, studies have not revealed the significant effects of Cu fertilization on strawberry yield [12,50]. In strawberries, a foliar concentration of Cu that is less than 5 to 6 mg kg^−1^ of dry matter is associated with deficiency [17,26]. During the sampling period from 2014 to 2016, more than 90% of strawberry leaf samples in Latvia were low in Cu, with a mean concentration of 3.97 ± 0.21 mg kg^−1^. An improvement in Cu supply was detected over the following years when the average Cu content in the leaves reached the lower end of the recommended range of 6–20 mg kg^−1^. At the same time, the percentage of deficient samples also decreased from 90% to 50%. As Cu provides a natural fungicidal effect, Cu products are often used in strawberry production as plant protection agents. This also has a beneficial effect on the Cu content in the leaves.

Similar to Zn, statistically significant positive correlations were also found between Cu and N, P, K, and S (0.372 > *r* < 0.524, *p* < 0.05) in strawberry leaves. Additionally, Cu was positively associated with Zn. Prasad et al. [46] also reported the synergy of Cu and N in strawberries, thus confirming the importance of Cu in N uptake and vice versa.

Overall, plants need a set of essential nutrients to synthesize their constituent compounds and for essential metabolic reactions. In addition, a balanced supply is a very important factor for increasing crop yield. Positive (synergism) and negative (antagonism) interactions between the nutrients are well-known and studied phenomena in plants, although there are still a lot of uncertainties [25]. Understanding nutrient interactions are also important in commercial crop production, as it can lead to more rational fertilization practices, with the timely prevention of interactions that could contribute to imbalances. In most cases, antagonism is related to the excess of one nutrient in the soil or in a plant, which interferes with the absorption of another element, its inclusion in the metabolism, or its uptake in a specific plant organ [51]. The antagonistic effect of K on Ca and Mg accumulation is well-known in vegetable and fruit production. Thus, K/Ca antagonism is one of the reasons for the high risk of physiological Ca disorders such as blossom-end rot (BER) [52]. An inhibitory effect of the ammonium form of N on Ca uptake was also reported [53].

The application of high doses of P fertilizers is often associated with Zn and Fe deficiency in plants due to the formation of poorly soluble Zn and Fe phosphates [51,54]. The significant decrease in Zn and Fe uptake from the soil is the key factor explaining the antagonistic interaction between these nutrients. However, internal immobilization is also possible. P-induced micronutrient (Fe, Cu, Mn, Zn) deficiency was also reported in strawberries [12,55]. In addition, the Fe deficiency-induced chlorosis of strawberry leaves could be more related to high Ca content in the soil than to Fe deficiency [12].

The participation of different elements in the same compounds or key processes in plants are the underlying mechanisms of synergistic interactions when a better supply of one nutrient promotes the absorption of another [25]. The positive interaction between N and S is mainly explained by their participation in protein synthesis [56], between N and P—by increasing root growth and soil acidity [51]. A positive interaction between Ca and B was demonstrated in strawberries [57], mainly due to the important role of both nutrients in cell wall strengthening.

In general, interactions between nutrients depend largely on the severity of nutrient deficiency or excess. In conditions where there are no pronounced nutrient imbalances in the soil, positive correlations between nutrients in the plant most likely indicate that better-supplied plants are more vital, with a better-developed root system, and thus, are able to accumulate nutrients more efficiently. This was largely confirmed by the results of our study on commercially grown strawberries in Latvia when all statistically significant correlations between the leaf nutrients were positive, and no drastic imbalances in fertilization management were found.

In general, strawberry cultivation has long traditions in Latvia. Strawberries have been grown commercially since the end of the 19th century [58]. Since climatic factors promote the formation of highly aromatic berries, strawberries grown in Latvia are out of competition in the local market during their harvesting season. Unfortunately, in the last two decades, the average annual production decreased more than two times compared to 1992–2001, when an average of 5.990 tons was produced [1]. Based on current trends, a significant increase in strawberry production was not predicted in Latvia and other Baltic States. Therefore, scientists have significant challenges in solving current cultivation problems in order to increase yield and provide consumers with fresh local berry production. Commercial strawberry production requires intensive and precise fertilization throughout the season to ensure optimal growth, fruit quality, and economic returns. In this aspect, the results of our research revealed several imbalances in the supply of nutrients—Ca, S, Zn, Cu deficiency in strawberry leaves, which could have a significant negative impact on strawberry production in Latvia.

## 3. Materials and Methods

### 3.1. Study Site and Sampling

Latvia is located in the hemiboreal climate zone. The climate is mild and humid, with four distinct seasons. The average annual precipitation in Latvia is 685.6 mm. The rainiest months are July and August, with an average of 76.3 mm of precipitation per month. Air temperature is seasonal—the coldest month is February, with an average air temperature of −3.1 °C, and the warmest is July, with +17.8 °C [59]. Such climatic conditions are generally suitable for strawberry cultivation [15].

In order to find out the nutrient status of strawberries that are commercially grown in Latvia, the results of 200 strawberry leaf samples from different strawberry-producing farms throughout Latvia were analysed over 3 periods: 2014–2016, 2017–2019, and 2020–2022. Leaf samples were taken and supplied by growers during the production season from June to August. The leaf samples consisted of 20–40 most recently mature trifoliate leaves from the uniform area. The content of the nutrients was analysed at the Laboratory of Plant Mineral Nutrition of the Institute of Biology, University of Latvia. Although strawberry cultivars were not always reported by the growers, June-bearing cultivars ‘Elsanta’, ‘Polka’, ‘Asia’, ‘Sonata’, ‘Rumba’, and ‘Malling Centenary’ were found to be the most common.

### 3.2. Nutrient Analysis

Air-dried leaf samples (leaf blades) were ground and dry-ashed in concentrated HNO_3_ vapours, and the ash was dissolved in a 3% HCl solution. Wet digestion in H_2_SO_4_ and HNO_3_ was used for N and S detection, respectively. Atomic absorption spectroscopy, using an acetylene-air flame atomizer (Perkin Elmer AAnalyst 700) and microwave plasma atomic emission spectrometry (4200 MP-AES, Agilent), was used for the measurement of K, Ca, Mg, Fe, Mn, Zn, and Cu according to the manufacturer’s instructions. Levels of P, Mo, N, and B were determined by colorimetry: P by ammonium molybdate in an acid-reduced medium, Mo by thiocyanate in a reduced acid medium, B by hinalizarine in a sulphuric acid medium, N by modified Kjeldal method using Nessler’s reagent in an alkaline medium, and S through the turbidimetric method by adding BaCl_2_, using a spectrophotometer Jenway 6300 as described previously [60]. All the values were expressed as mass % and mg kg^−1^ on a dry matter basis for each macronutrient and micronutrient evaluated, respectively.

### 3.3. Statistical Analysis

Data on the nutrient content of strawberry leaves were analysed with descriptive statistics. Standard errors (SE) were calculated to reflect the mean results of nutrient concentrations. The heterogeneity of leaf nutrient concentrations was characterized by the coefficient of variation (CV). Student’s *t*-test and ‘Two-Sample Assuming Unequal Variances’ (*p* < 0.05) with Bonferroni correction was used to check the significance of the differences in the nutrient status of strawberries between the study periods. The correlation analysis was determined using Pearson’s correlation analysis.

## 4. Conclusions

This study has provided insight into clarifying whether the actual nutrient status and possible imbalances in nutrient supply could significantly affect the strawberry yield in Latvia. According to the leaf analyses, plant fertilization was only partly well managed by the growers. Although a positive trend was found in the supply of strawberries with nutrients: the percentage of the total optimal concentrations in leaves increased from 54% in 2014–2016 to 67% in 2020–2022, more than a third of the indices did not reach desired levels. In the period from 2014 to 2022, strawberries in Latvia were generally sufficiently supplied with N, P, K, Mg, Fe, Mn, Mo, and B, while the supply of Ca, S, Zn, and Cu was considered low. The deficiency of these nutrients was characteristic for more than 50% of the samples. As Ca, S, Zn, and Cu are essential for plant growth and metabolism and contribute to stress resistance, berry formation, and quality: their deficiency could be one of the limiting factors affecting strawberry productivity in Latvia. Therefore, special attention should be paid to the adequate provision of Ca, S, Zn, and B. Considering the low availability of information on the relationship between nutrient supply and strawberry yield in the Baltic region, as well as the significant economic potential of strawberries, further research in this direction needs to be performed.

## Figures and Tables

**Table 1:** Nutrient concentrations in air-dry strawberry leaves from commercial plantings in Latvia, 2014–2022.

	2014–2016 *n* = 46		2017–2019 *n* = 65		2020–2022 *n* = 89		Nutrient Sufficiency Range ^2^
	Mean ± SE Range	CV ^1^	Mean ± SE Range	CV	Mean ± SE Range	CV	
Macronutrients, %							
N	2.44 ± 0.10 a ^3^	27.23	2.32 ± 0.08 a	27.86	2.62 ± 0.06 a	21.00	2.0–3.0
	1.33–4.07		1.05–3.60		1.30–4.70		
P	0.30 ± 0.01 a	31.00	0.30 ± 0.01 a	32.19	0.40 ± 0.01 b	30.11	0.2–0.4
	0.18–0.56		0.17–0.68		0.20–0.78		
K	1.67 ± 0.06 a	26.27	1.55 ± 0.06 a	29.15	1.70 ± 0.05 a	28.37	1.5–2.5
	0.74–3.10		0.84–2.66		0.80–2.91		
Ca	0.70 ± 0.06 a	60.00	0.68 ± 0.03 a	37.75	0.73 ± 0.03 a	35.39	0.7–2.0
	0.20–1.82		0.23–1.40		0.34–1.72		
Mg	0.32 ± 0.01 a	31.03	0.32 ± 0.01 a	25.29	0.28 ± 0.01 a	24.39	0.2–0.5
	0.18–0.54		0.19–0.50		0.17–0.47		
S	0.13 ± 0.01 a	35.99	0.12 ± 0.01 a	34.05	0.15 ± 0.01 a	42.84	0.2–0.8
	0.05–0.34		0.06–0.28		0.07–0.50		
Micronutrients, mg kg^−1^							
Fe	83.7 ± 5.3 a	43.09	88.1 ± 6.0 a	55.16	85.9 ± 4.2 a	47.77	50–250
	44–184		46–358		41–230		
Mn	56.3 ± 6.1 a	73.94	77.5 ± 5.8 b	60.71	74.9 ± 8.2 b	102.88	25–200
	10.6–204.0		17–248		7.6–480		
Zn	18.1 ± 0.9 a	32.82	18.6 ± 0.8 a	34.13	21.6 ± 0.9 b	37.03	20–50
	9.8–36.0		804–40.0		9.0–44.0		
Cu	3.97 ± 0.21 a	35.32	5.36 ± 0.20 b	29.96	5.93 ± 0.25 b	39.29	6–20
	2.0–10.2		1.0–9.2		2.8–14.7		
Mo	1.05 ± 0.28 a	177.67	0.78 ± 0.08 a	86.95	1.17 ± 0.15 a	122.05	>0.5
	0.18–10.2		0.25–4.8		0.20–10.80		
B	24.4 ± 1.4 a	39.70	29.1 ± 1.3 b	34.79	38.2 ± 2.5 b	60.55	20–70
	10–63		10–54		9–180		

^1^ Coefficient of variation. ^2^ Established nutrient sufficiency range for healthy strawberry leaves [15,16,17,18]. ^3^ Means with different letters in a row were significantly different between study periods (*t*-test, *p* < 0.05, a < b).

**Table 2:** Pearson’s correlation matrix between nutrient concentrations in strawberry leaves in Latvia, 2014–2022.

	N	P	K	Ca	Mg	S	Fe	Mn	Zn	Cu	Mo
P	0.675 *										
K	0.567 *	0.639 *									
Ca	−0.024	0.016	−0.028								
Mg	0.338 *	0.329 *	0.311 *	0.650 *							
S	0.566 *	0.552 *	0.419 *	−0.035	0.271 *						
Fe	0.259 *	0.165	0.002	0.081	0.163	0.044					
Mn	0.136	0.104	0.108	0.116	0.078	0.024	0.148				
Zn	0.648 *	0.680 *	0.607 *	−0.059	0.255 *	0.536 *	0.192 *	0.275 *			
Cu	0.419 *	0.524 *	0.311 *	−0.088	0.085	0.372 *	0.096	0.097	0.508 *		
Mo	0.139	0.167	0.084	0.284 *	0.202 *	0.031	0.172	0.142	0.086	−0.112	
B	0.219 *	0.321 *	0.253 *	0.319 *	0.200 *	0.259 *	0.116	0.190 *	0.165	0.166	0.297 *

The colour intensity of squares is proportional to Pearson’s correlation coefficients, red colour indicates positive correlations and blue—negative correlations. Asterisk (*) indicates significance at *p* < 0.01, *r* > 0.181, *n* = 200.

## Data Availability

All data reported here are available from the authors upon request.

## References

[1] Global Trends, Statistics and Insights for Strawberry. https://www.nationmaster.com/nmx/sector/strawberry.

[2] Agriculture of Latvia (2022). Collection of Statistics.

[3] Kalniņa I., Strautiņa S. Analysis of climatic factors in connection with strawberry generative bud development. Proceedings of the International Scientific Conference Research for Rural Development.

[4] Kalnina I., Sterne D., Strautina S. (2016). Strawberry (*Fragaria ananassa*) cv. ‘Rumba’ assessment under the northern climatic conditions. Acta Hortic..

[5] Kalniņa I., Strautiņa S., Laugale V. (2019). Strawberry ‘Flair’ and ‘Felicita’ suitability for forcing under high tunnel. Acta Hortic..

[6] Kalnina I., Strautina S., Silina L., Laugale V. (2014). The possibilities of strawberry growing under high tunnels in Latvia. Acta Hortic..

[7] Laugale V., Dane S., Lepse L., Strautina S., Kalnina I. (2017). Influence of low tunnels on strawberry production time and yield. Acta Hortic..

[8] Domínguez A., Martínez F., Allendes G., Palencia P. (2020). Evaluation of the nutritional status of strawberry during the production season. Environ. Eng. Manag. J..

[9] Nestby R., Lieten F., Pivot D., Raynal Lacroix C., Tagliavini M. (2005). Influence of mineral nutrients on strawberry fruit quality and their accumulation in plant organs. Int. J. Fruit Sci..

[10] Soppelsa S., Kelderer M., Casera C., Bassi M., Robatscher P., Matteazzi A., Andreotti C. (2019). Foliar applications of biostimulants promote growth, yield and fruit quality of strawberry plants grown under nutrient limitation. Agronomy.

[11] Duralija B., Mikec D., Jurić S., Lazarević B., Maslov Bandić L., Vlahoviček-Kahlina K., Vinceković M. (2021). Strawberry fruit quality with the increased iron application. Acta Hortic..

[12] Trejo-Téllez L.I., Gómez-Merino F.C., Malone N. (2014). Nutrient management in strawberry: Effects on yield, quality and plant health. Strawberries: Cultivation, Antioxidant Properties and Health Benefits.

[13] Medeiros R.F., Pereira W.E., Rodrigues R.M., Nascimento R., Suassuna J.F., Dantas T.A.G. (2015). Growth and yield of strawberry plants fertilized with nitrogen and phosphorus. Rev. Bras. Eng. Agric. Ambient..

[14] Shirko R., Nazarideljou M.J., Akba M.A., Naser G. (2018). Photosynthetic reaction, mineral uptake, and fruit quality of strawberry affected by different levels of macronutrients. J. Plant Nutr..

[15] Nutritional Recommendations for Strawberry. https://www.haifa-group.com/files/Guides/Strawberry/strawberry.pdf.

[16] Bottoms T.G., Bolda M.P., Gaskell M.L., Hartz T.K. (2013). Determination of strawberry nutrient optimum ranges through diagnosis and recommendation integrated system analysis. Horttechnology.

[17] Pritts M.P. (2015). Nutrient management practices in perennial strawberry are informed by understanding the relationships among carbohydrate status, nitrogen availability, and soil composition. Horttechnology.

[18] Dixon E., Strik B., Fernandez-Salvador J., DeVetter L.W. (2019). Strawberry Nutrient Management Guide for Oregon and Washington.

[19] Niskanen R., Dris R. (2002). Nutritional status of strawberry fields. Acta Hortic..

[20] Kimptom T. (2016). Determine Optimum Nitrogen and Potassium Requirement to Maximise Yield and Quality of Day-Neutral Victorian Strawberries.

[21] Nollendorfs V. (2003). Strawberry fertilization. Agrotops.

[22] Sprogis K., Kince T., Muizniece-Brasava S. Investigation of fertilisation impact on fresh strawberries yield and quality parameters. Proceedings of the 11th Baltic Conference on Food Science and Technology “Food Science and Technology in a Changing World”.

[23] Laugale V., Dane S., Lepse L., Strautiņa S. (2017). Fruit quality and resistance of strawberry cultivars and hybrids and the effect of calcite fertilizer. Proc. Latv. Acad. Sci. Sect. B.

[24] Laugale V., Dane S., Strautina S., Kalnina I. (2020). Influence of wermicompost on strawberry plant growth and dehydrogenase activity in soil. Agron. Res..

[25] Marschner P. (2012). Mineral Nutrition of Higher Plants.

[26] Hochmuth G., Albregts E. (2019). Fertilization of Strawberries in Florida.

[27] Narayan O.P., Kumar P., Yadav B., Dua M., Johri A.K. (2022). Sulfur nutrition and its role in plant growth and development. Plant Signal. Behav..

[28] Tripathi R., Tewari R., Singh K.P., Keswan i.C., Minkina T., Srivastava A.K., De Corato U., Sansinenea E. (2022). Plant mineral nutrition and disease resistance: A significant linkage for sustainable crop protection. Front. Plant Sci..

[29] Campbell C.R., Miner G.S., Campbell C.R. (2000). Strawberry, annual hill culture. Reference Sufficiency Ranges for Plant Analysis in the Southern Region of the United States.

[30] Hick K. Optimize Strawberry Fertility with Plant Tissue Testing. https://smallfruits.org/2022/04/optimize-strawberry-fertility-with-plant-tissue-testing/.

[31] Jamal A., Moon Y., Abdin M.Z. (2010). Sulphur—A general overview and interaction with nitrogen. Aust. J. Crop Sci..

[32] Liu S., Cui S., Zhang X., Wang Y., Mi G., Gao Q. (2020). Synergistic regulation of nitrogen and sulfur on redox balance of maize leaves and amino acids balance of grains. Front. Plant Sci..

[33] Zenda T., Liu S., Dong A., Duan H. (2021). Revisiting sulphur—The once neglected nutrient: It’s roles in plant growth, metabolism, stress tolerance and crop production. Agriculture.

[34] Santos B.M. (2013). Response of strawberries to preplant sulphur fertilization in sandy soils. Int. J. Fruit Sci..

[35] Aas W., Mortier A., Bowersox V., Cherian R., Faluvegi G., Fagerli H., Hand J., Klimont Z., Galy-Lacaux C., Lehmann C.M.B. (2019). Global and regional trends of atmospheric sulfur. Sci. Rep..

[36] Singh R., Sharma R.R., Tyagi S.K. (2007). Pre-harvest foliar application of calcium and boron influences physiological disorders, fruit yield and quality of strawberry. Sci. Hortic..

[37] Valentinuzzi F., Mason M., Scampicchio M., Andreotti C., Cesco S., Mimmo T. (2015). Enhancement of the bioactive compound content in strawberry fruits grown under iron and phosphorus deficiency. J. Sci. Food Agric..

[38] Bieniasz M., Małodobry M., Dziedzic E. (2012). The effect of foliar fertilization with calcium on quality of strawberry cultivars ‘Luna’ and ‘Zanta’. Acta Hortic..

[39] Sidhu R.S., Singh N.P., Singh S., Sharda R. (2020). Foliar nutrition with calcium nitrate in strawberries (*Fragaria × ananassa* Duch.): Effect on fruit quality and yield. Indian J. Ecol..

[40] Vance A.J., Jones P., Strik B.C. (2017). Foliar calcium applications do not improve quality or shelf life of strawberry, raspberry, blackberry, or blueberry fruit. Hortscience.

[41] Alloway B.J. (2009). Soil factors associated with zinc deficiency in crops and humans. Environ. Geochem. Health.

[42] Valujeva K., Nipers A., Lupikis A., Schulte R.P.O. (2020). Assessment of soil functions: An example of meeting competing national and international obligations by harnessing regional differences. Front. Environ. Sci..

[43] López-Herrera A., Castillo-González A.M., Trejo-Téllez L.I., Avitia-García E., Valdez-Aguilar L.A. (2018). Strawberry response cv. Albion at increasing doses of zinc. Rev. Mex. Cienc. Agríc..

[44] Bhatti S.M., Panhwar M.A., Bughio Z.R., Sarki M.S., Gandahi A.W., Wahocho N.A. (2021). Influence of foliar application of zinc on growth, yield and zinc concentration in strawberry. Pakistan J. Agri. Res..

[45] Mahmood M.M., Al-Dulaimy A.F. (2021). Response of strawberry CV Festival to culture media and foliar application of nano and normal micronutrients. IOP Conf. Ser. Earth Environ. Sci..

[46] Prasad R., Lisiecka J., Kleiber T. (2022). Morphological and yield parameters, dry matter distribution, nutrients uptake, and distribution in strawberry (*Fragaria × ananassa* Duch.) cv. ‘Elsanta’ as influenced by spent mushroom substrates and planting seasons. Agronomy.

[47] Tohidloo G., Souri M.K., Eskandarpour S. (2018). Growth and fruit biochemical characteristics of three strawberry genotypes under different potassium concentrations of nutrient solution. Open Agric..

[48] Salman M., Ullah S., Razzaq K., Rajwana I.A., Akhtar G., Faried H.N., Hussain A., Amin M., Khalid S. (2022). Combined foliar application of calcium, zinc, boron and time influence leaf nutrient status, vegetative growth, fruit yield, fruit biochemical and anti-oxidative attributes of “Chandler” strawberry. J. Plant Nutr..

[49] Evans I., Solberg E., Huber D.M., Datnoff L.E., Elmer W.H., Huber D.M. (2007). Copper and plant diseases. Mineral Nutrition and Plant Disease.

[50] Sabahat S., Abbasi J., Mumtaz S., Tariq S., Imran M., Ahmad M., Khan T.N. (2021). Role of micronutrients in improving fruit quality and yield of strawberry cv. Chandler under microclimatic conditions. Pak. J. Agri. Res..

[51] Fageria V.D. (2001). Nutrient interactions in crop plants. J. Plant Nutr..

[52] Fanasca S., Rouphael Y., Cardarelli M., Colla G. (2005). The influence of K:Ca:Mg:Na ratio and total concentration on yield and fruit quality of soilless-grown tomatoes: A modelling approach. Acta Hortic..

[53] Bonomelli C., de Freitas S.T., Aguilera C., Palma C., Garay R., Dides M., Brossard N., O’Brien J.A. (2021). Ammonium excess leads to Ca restrictions, morphological changes, and nutritional imbalances in tomato plants, which can be monitored by the N/Ca ratio. Agronomy.

[54] Yu B.G., Chen X.X., Cao W.Q., Liu Y.M., Zou C.Q. (2020). Responses in zinc uptake of different mycorrhizal and non-mycorrhizal crops to varied levels of phosphorus and zinc applications. Front. Plant Sci..

[55] Choi J.M., Lee C.W. (2012). Influence of elevated phosphorus levels in nutrient solution on micronutrient uptake and deficiency symptom development in strawberry cultured with fertigation system. J. Plant Nutr..

[56] Astolfi S., Celletti S., Vigani G., Mimmo T., Cesco S. (2021). Interaction between sulfur and iron in plants. Front. Plant Sci..

[57] Wójcik P., Lewandovski M. (2003). Effect of calcium and boron sprays on yield and quality of “Elsanta” strawberry. J. Plant Nutr..

[58] Kaufmane E., Skrīvele M., Rubauskis E., Strautiņa S., Ikase L., Lācis G., Segliņa D., Moročko-Bičevska I., Ruisa S., Priekule I. (2013). Development of fruit science in Latvia. Proc. Latvian Acad. Sci. Sect. B.

[59] SLLC “Latvian Environment, Geology and Meteorology Centre” Latvijas Klimats. https://videscentrs.lvgmc.lv/lapas/latvijas-klimats.

[60] Cekstere G., Osvalde A., Elferts D., Rose C., Lucas F., Vollenweider P. (2020). Salt accumulation and effects within foliage of Tilia x vulgaris trees from the street greenery of Riga, Latvia. Sci. Total Environ..

